# Selective elimination of quiescent cancer cells by dequalinium chloride via ROS‐mediated ferroptosis

**DOI:** 10.1002/2211-5463.70344

**Published:** 2026-10-05

**Authors:** Kotaro Miyamoto, Shiori Sakai, Minori Endo, Terigele, Mizuho Niibori, Taiki Morita, Yu Goto, Arisa Yoshida, Tomohiro Tanaka, Ning Lin, Takahiro Kuchimaru, Hiroyuki Nakamura, Masahiro Inoue, Shinae Kizaka‐Kondoh, Tetsuya Kadonosono

**Affiliations:** ^1^ School of Life Science and Technology Institute of Science Tokyo Yokohama Japan; ^2^ Institute of Integrated Research Institute of Science Tokyo Yokohama Japan; ^3^ Center for Molecular Medicine Jichi Medical University Shimotsuke Japan; ^4^ Graduate School of Medicine Kyoto University Japan; ^5^ Osaka Medical Center for Cancer and Cardiovascular Diseases Japan; ^6^ National Institute of Technology, Nara College Yamatokoriyama Japan

**Keywords:** dequalinium chloride, ferroptosis, mitochondria, quiescent cancer cells, reactive oxygen species

## Abstract

Quiescent cancer cells (QCCs) drive tumor dormancy and recurrence through their resistance to conventional therapies. Furthermore, because QCCs rely on oxidative phosphorylation and maintain a fragile redox balance, we hypothesized that they harbor a targetable redox vulnerability. Here, we show that the mitochondria‐targeted agent dequalinium chloride (DQ) selectively kills QCCs in hypoxia‐ and nutrient limitation‐induced quiescent models, including H2228 cells, multicellular spheroids, and colorectal cancer organoids, while sparing proliferating cells and normal fibroblasts. DQ induces ROS accumulation beyond the survival threshold of QCCs, leading to lipid peroxidation and ferroptotic cell death, whereas proliferating cells undergo limited caspase‐independent apoptosis. These findings highlight redox imbalance as an actionable vulnerability of QCCs and position DQ as a potential repurposed agent for eliminating QCCs.

## Graphical abstract

Dequalinium chloride (DQ) selectively eliminates quiescent cancer cells (QCCs) by amplifying mitochondrial ROS. QCCs exhibit elevated basal ROS and limited redox‐buffering capacity, making them vulnerable to further ROS increases. DQ‐induced ROS exceeds the tolerance threshold, triggering lipid peroxidation and ferroptosis, whereas proliferating cells remain less sensitive.

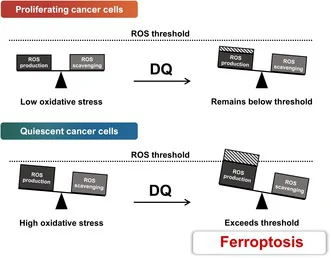

**Abbreviations**
DQ: dequalinium chloride
FCM: flow cytometry
GG: general growth
HGD: hypoxic and growth supplement‐deprived
HLS: hypoxic and low‐serum
MCS: multicellular spheroid
OXPHOS: oxidative phosphorylation
QCC: quiescent cancer cells
ROS: reactive oxygen species
TME: tumor microenvironment

Quiescent cancer cells (QCCs) are a non‐proliferative subpopulation that can survive stresses within the tumor microenvironment (TME) [1] and contribute to tumor dormancy, relapse, and therapy resistance [2]. Because conventional anticancer therapies primarily target rapidly dividing cells [3], QCCs can persist after treatment, re‐enter the cell cycle, and drive minimal residual disease and late recurrence [2, 4]. Therefore, developing strategies to eliminate QCCs prior to their reactivation is of significant clinical importance.

QCCs enter a reversible cell‐cycle arrest regulated by extracellular matrix signals, niche‐derived factors, and hypoxic and nutrient‐limited conditions within the TME [5, 6], and resume proliferation upon receiving appropriate cues [7]. During quiescence, QCCs undergo metabolic and epigenetic adaptations and evade immune surveillance, enabling long‐term persistence [1, 8]. Under hypoxic and nutrient‐limited conditions, QCCs rely predominantly on oxidative phosphorylation (OXPHOS) rather than glycolysis and exhibit high metabolic plasticity [9, 10, 11]. This OXPHOS dependence is a metabolic hallmark of QCCs and represents a potential therapeutic vulnerability.

Mitochondrial reactive oxygen species (ROS), byproducts of OXPHOS, are normally neutralized by antioxidant systems [12, 13]. When ROS production exceeds antioxidant capacity, redox homeostasis collapses, triggering programmed cell death pathways including caspase‐dependent apoptosis [14, 15], EndoG‐mediated caspase‐independent apoptosis [16], and ferroptosis [17]. QCCs within the TME maintain elevated basal ROS associated with sustained OXPHOS [18, 19] while simultaneously activating ROS‐scavenging mechanisms [20]. This stressed redox state may render QCCs particularly susceptible to further ROS elevation, suggesting that pharmacological ROS induction could selectively eliminate QCCs [21].

Ferroptosis is an iron‐dependent form of regulated cell death driven by lipid peroxidation [22, 23]. GPX4 is a key antioxidant enzyme that suppresses ferroptosis by reducing phospholipid hydroperoxides [17, 24]. Heme oxygenase‐1 (HO‐1), encoded by HMOX1, links redox homeostasis to iron metabolism through heme degradation. Although NRF2‐associated HMOX1 induction can protect cells against oxidative stress, excessive HO‐1 activity can increase labile iron and promote ferroptosis [24].

Dequalinium chloride (DQ), a cationic amphiphilic molecule clinically used as an antimicrobial agent [25, 26], accumulates in mitochondria, where it disrupts membrane potential, suppresses ATP synthesis [27, 28], increases ROS levels, and induces oxidative stress [29, 30]. Although DQ has been reported to induce multiple forms of cell death in proliferating cancer cells [31, 32], its effects on QCCs remain undefined. Given its mitochondrial tropism and ability to induce oxidative stress, we hypothesized that DQ may preferentially eliminate QCCs by exacerbating ROS accumulation and triggering ferroptosis. In this study, using hypoxia‐ and nutrient limitation‐induced quiescent H2228 non‐small cell lung cancer (NSCLC) cells, we show that DQ preferentially kills QCCs by inducing ROS‐dependent ferroptosis.

## Materials and methods

### Ethics statement

Experiments using human organoids were approved by the Institutional Ethics Committees of the Institute of Science Tokyo (approval number 2014086). The surgical specimen used to establish the C45 organoids was obtained with the donor's understanding and written informed consent. All study methodologies involving human material conformed to the standards set by the Declaration of Helsinki.

### Cancer organoids and cell lines

Detailed methods for cancer organoid preparation by the cancer tissue‐originated spheroid (CTOS) method and expansion have been described previously [33]. The human colorectal cancer organoid line C45 was cultured in StemPro hESC SFM (Thermo Fisher Scientific, Waltham, MA, USA) at 37 °C in 5% CO_2_. The medium was partially replaced every 2–3 days, and organoids were maintained in suspension culture. Before use, organoids were mechanically sheared using a 27‐gauge needle and cultured for 1 day.

The lung cancer cell line NCI‐H2228 (CRL‐5935; RRID:CVCL_1543) and the normal human embryonic lung fibroblast cell line WI‐38 (RCB0704; RRID:CVCL_0579) were obtained from the American Type Culture Collection (ATCC, Manassas, VA, USA) and the RIKEN BioResource Research Center (Tsukuba, Japan), respectively. H2228 and WI‐38 cells were maintained in RPMI1640 or DMEM supplemented with 10% FBS (Thermo Fisher Scientific, USA), respectively, at 37 °C in 5% CO_2_. Penicillin (100 U/mL) and streptomycin (100 μg/mL) (Nacalai Tesque, Kyoto, Japan) were added to the media. Cells were routinely tested for mycoplasma contamination using a mycoplasma detection kit (Lonza, Basel, Switzerland), and all experiments were performed using cells confirmed to be mycoplasma‐free. Cells were thawed from original stocks for each experiment. Multicellular spheroids (MCSs) were generated by culturing H2228 cells (4.0 × 10^5^ cells) in non‐coated 9‐cm polystyrene dishes for 7 days.

H2228 and WI‐38 cells were authenticated within the past 3 years by short tandem repeat profiling performed by ATCC and RIKEN BioResource Research Center. The resulting profiles matched the corresponding reference profiles.

### Proliferation assay

Cells were cultured under hypoxic conditions in a multigas incubator (ASTEC, Fukuoka, Japan). H2228 cells (2.0 × 10^4^ cells/well) were seeded in 6‐well plates and cultured for 6 days under hypoxic and low‐serum (HLS; 1% O_2_, 5% CO_2_, and 0.1% FBS‐RPMI1640) or general growth (GG; 21% O_2_, 5% CO_2_, and 10% FBS‐RPMI1640) conditions. H2228 MCSs (10–30 spheroids/well) were seeded in 96‐well plates and cultured for 6 days under HLS (1% O_2_, 5% CO_2_, and 0.8% FBS‐RPMI1640) or GG (21% O_2_, 5% CO_2_, and 10% FBS‐RPMI1640) conditions. C45 organoids (10–30 spheroids/well) were seeded in 96‐well plates and cultured for 6 days under hypoxic and growth supplement‐deprived (HGD; 1% O_2_, 5% CO_2_, and StemPro hESC SFM without growth supplement) or GG (21% O_2_, 5% CO_2_, and StemPro hESC SFM with growth supplement) conditions.

Living H2228 cells were labeled with CellTracker Green CMFDA (Thermo Fisher Scientific, USA) and counted using a BZ‐X810 microscope (Keyence, Osaka, Japan). The volumes of H2228 MCSs and C45 organoids were calculated from spheroid diameters. Relative cell number or relative volume was calculated by normalizing values to Day 0.

### DQ‐D synthesis

The DQ derivative, 4‐amino‐1‐decyl‐2‐methylquinolin‐1‐ium iodide (DQ‐D), was synthesized from 2‐methylquinolin‐4‐amine and 1‐iododecane. A mixture of 2‐methylquinolin‐4‐amine (316 mg, 2.00 mmol, 1.00 equiv.) and 1‐iododecane (1.27 mL, 6.00 mmol, 3.00 equiv.) was dissolved in acetonitrile (10 mL) in a sealed vial under argon and heated at 150 °C for 2 h under microwave irradiation (300 W). After cooling, the precipitate was collected by vacuum filtration, washed with tetrahydrofuran, and dried *in vacuo* to obtain a white solid. The structure of DQ‐D was confirmed by NMR and mass spectrometry using an AVANCE III NMR spectrometer (Bruker, Billerica, MA, USA), a JMS‐700 MStation mass spectrometer (JEOL, Tokyo, Japan), and a Bruker micrOTOF II (Bruker, USA).

### EC_50_ measurement

Quiescent H2228 cells (1.0 × 10^3^ cells/well), H2228 MCSs (10–30 spheroids/well), and C45 organoids (10–30 spheroids/well) were prepared in 96‐well plates by culture under HLS or HGD conditions for at least 4 days. Serial concentrations of DQ (FUJIFILM Wako Pure Chemical, Osaka, Japan), DQ‐D, or rotenone (MedChemExpress, Monmouth Junction, NJ, USA) were then added, and samples were incubated for 1 or 3 days under GG, HLS, or HGD conditions. Cytotoxicity was evaluated by measuring the number of living H2228 cells using Calcein‐AM dye (Dojindo, Kumamoto, Japan) and by counting spheroids in H2228 MCSs and C45 organoids. EC_50_ values were calculated using GraphPad Prism software (GraphPad Software, Boston, MA, USA).

### Cytotoxicity assay

WI‐38 cells (2.0 × 10^4^ cells) were cultured in 6‐well plates under HLS or GG conditions and treated with 1 μm DQ for 1 day. Quiescent or proliferating H2228 cells (2.0 × 10^4^ cells), cultured under HLS or GG conditions, respectively, were treated with 1 μm DQ in the presence or absence of 10 μm ferrostatin‐1 (Cayman Chemical, MI, USA) for 3 days. Cytotoxicity was evaluated by staining living cells with CellTracker Green CMFDA and counting them using Fiji [34].

### Membrane potential analysis

Quiescent or proliferating H2228 cells (1.0 × 10^5^ cells) cultured in 10‐cm dishes under HLS or GG conditions, respectively, were treated with 0, 0.2, or 0.5 μm DQ for 3 days. Cells were incubated with 1 μg/mL rhodamine 123 (FUJIFILM Wako Pure Chemical, Japan) for 15 min, collected by trypsinization, washed with PBS, and analyzed using an iCyt ec800 flow cytometer (Sony Biotechnology, San Jose, CA, USA).

### Intracellular ATP measurement

Quiescent or proliferating H2228 cells (1.0 × 10^5^ cells) cultured in 10‐cm dishes under HLS or GG conditions, respectively, were treated with 0, 0.2, or 0.5 μm DQ for 1 day. Cells were collected by trypsinization, and intracellular ATP levels were measured using an intracellular ATP assay kit (Toyo B‐Net, Tokyo, Japan).

### ROS measurement

Quiescent or proliferating H2228 cells (1.0 × 10^5^ cells) cultured in 10‐cm dishes under HLS or GG conditions, respectively, were treated with 0, 0.2, or 0.5 μm DQ or rotenone for 3 days. Cells were incubated with 10 μm H_2_DCFDA (Thermo Fisher Scientific, USA) for 30 min, collected by trypsinization, washed with PBS, and analyzed using a BD Accuri C6 Plus flow cytometer (BD Biosciences, Franklin Lakes, NJ, USA). Data were analyzed using FlowJo software (BD Biosciences, USA).

### GSSG/GSH quantification

GSSG/GSH ratios were measured using a GSSG/GSH Quantification kit (Dojindo, Japan) according to the manufacturer's instructions. Quiescent or proliferating H2228 cells (1.0 × 10^5^ cells) cultured in 10‐cm dishes under HLS or GG conditions, respectively, were treated with 0, 0.2, or 0.5 μm DQ for 3 days. Cells were lysed by two freeze–thaw cycles and mixed with 5% 5‐sulfosalicylic acid dihydrate (FUJIFILM Wako Pure Chemical, Japan). After centrifugation, the supernatant was analyzed by measuring the absorbance of 5,5′‐dithiobis(2‐nitrobenzoic acid) at 405 nm using a Model 680XR microplate reader (Bio‐Rad, Hercules, CA, USA).

### Cell death analysis

Quiescent or proliferating H2228 cells (1.0 × 10^5^ cells) cultured in 10‐cm dishes under HLS or GG conditions, respectively, were treated with 0, 0.2, or 0.5 μm DQ for 3 days and collected by scraping. Cells were stained with PE‐labeled Annexin V and 7‐AAD using a PE Annexin V Apoptosis Detection Kit I (BD Biosciences, USA) for 15 min, washed with 3% FBS in PBS, and analyzed using the iCyt ec800 flow cytometer.

### Caspase‐3/7 activity assay

Caspase‐3/7 activity was measured using a Caspase‐Glo 3/7 Assay System (Promega, Madison, WI, USA) according to the manufacturer's instructions. Proliferating H2228 cells (1.0 × 10^5^ cells) cultured in 10‐cm dishes under GG conditions were treated with 0, 0.2, or 0.5 μm DQ for 3 days or with 1 μm staurosporine (FUJIFILM Wako Pure Chemical, Japan) for 24 h. Cells were collected by trypsinization, incubated with Caspase‐Glo 3/7 reagent for 1 h, and luminescence was measured using a Fluoroskan FL microplate luminometer (Thermo Fisher Scientific, USA).

### Immunocytochemistry

Proliferating H2228 cells (2.0 × 10^4^ cells) cultured in removable 8‐well glass chambers (ibidi GmbH, Bavaria, Germany) under GG conditions were fixed with 4% paraformaldehyde in PBS for 15 min and permeabilized with 0.2% Triton X‐100 (Nacalai Tesque, Japan) in PBS for 10 min at room temperature. After washing with PBS containing 0.02% Tween 20, cells were blocked with PBS containing 2% FBS for 1 h and incubated overnight at 4 °C with anti‐human EndoG rabbit antibody (#22148, Proteintech, Rosemont, IL, USA). After washing, cells were incubated for 1 h at room temperature in the dark with Alexa Fluor 594‐conjugated goat anti‐rabbit IgG antibody (#A11012, Thermo Fisher Scientific). Nuclei were stained with bisbenzimide H33342 fluorochrome trihydrochloride (Nacalai Tesque, Japan). Images were acquired using the BZ‐X810 microscope. EndoG‐positive areas were quantified using Fiji software, and the percentage of nuclear EndoG was calculated as the ratio of nuclear EndoG‐positive area to total EndoG‐positive area.

### Lipid peroxide measurement

Quiescent or proliferating H2228 cells (1.0 × 10^5^ cells) cultured in 10‐cm dishes under HLS or GG conditions, respectively, were treated with 0, 0.2, or 0.5 μm DQ for 3 days. Cells were then incubated with 10 μm Liperfluo (Thermo Fisher Scientific, USA) for 30 min, collected by trypsinization, washed with 3% FBS in PBS, and analyzed using the BD Accuri C6 Plus flow cytometer. For inhibition of lipid peroxidation, quiescent H2228 cells cultured under HLS conditions were treated with 0 or 1 μm DQ in the presence or absence of 10 μm ferrostatin‐1 for 3 days.

### Quantitative real‐time PCR

Quiescent or proliferating H2228 cells (2.0 × 10^5^ cells) cultured in 6‐well plates under HLS or GG conditions, respectively, were treated with 0, 0.2, or 0.5 μm DQ for 3 days. Cells were collected by trypsinization, and total RNA was extracted using an RNeasy kit (Qiagen, Venlo, Netherlands). Quantitative real‐time PCR was performed using THUNDERBIRD® qPCR Mix (TOYOBO, Osaka, Japan) on a Thermal Cycler Dice® Real Time System III (Takara Bio, Shiga, Japan). Primer sequences were as follows: *GPX4* forward, 5′‐ACAAGAACGGCTGCGTGGTGAA‐3′ and reverse, 5′‐GCCACACACTTGTGGAGCTAGA‐3′; *HMOX1* forward, 5′‐CCAGGCAGAGAATGCTGAGTTC‐3′ and reverse, 5′‐AAGACTGGGCTCTCCTTGTTGC‐3′; and *B2M* forward, 5′‐CCACTGAAAAAGATGAGTATGCCT‐3′ and reverse, 5′‐CCAATCCAAATGCGGCATCTTCA‐3′. Relative gene expression was calculated using the 2^−ΔΔCt^ method, with *B2M* as the internal control.

### Intracellular labile Fe^2+^ measurement

Quiescent or proliferating H2228 cells (2.0 × 10^5^ cells) cultured in 6‐well plates under HLS or GG conditions, respectively, were treated with 0, 0.2, or 0.5 μm DQ for 3 days. Cells were incubated with 1 μm FerroOrange (Dojindo, Japan) for 30 min, collected by trypsinization, washed with 3% FBS in PBS, and analyzed using the BD Accuri C6 Plus flow cytometer.

### Statistical analyses

All experiments were performed independently at least three times. Data are presented as means ± standard error of the mean (SEM). Statistical significance was determined using a two‐sided Student's *t*‐test. A *P* value < 0.05 was considered statistically significant.

## Results

### DQ preferentially eliminates quiescent cancer cells

We employed two models that enter quiescence under hypoxic and nutrient‐limited conditions (Fig. 1A): H2228 lung cancer cells, cultured as monolayers (Fig. 1B) or MCSs (Fig. 1C), and C45 colorectal cancer organoids (Fig. 1D) [11, 33]. All models proliferated robustly under general growth (GG) conditions but entered quiescence under hypoxic and low‐serum (HLS) or hypoxic and growth supplement‐deprived (HGD) conditions (Fig. 1).

**Fig. 1:**
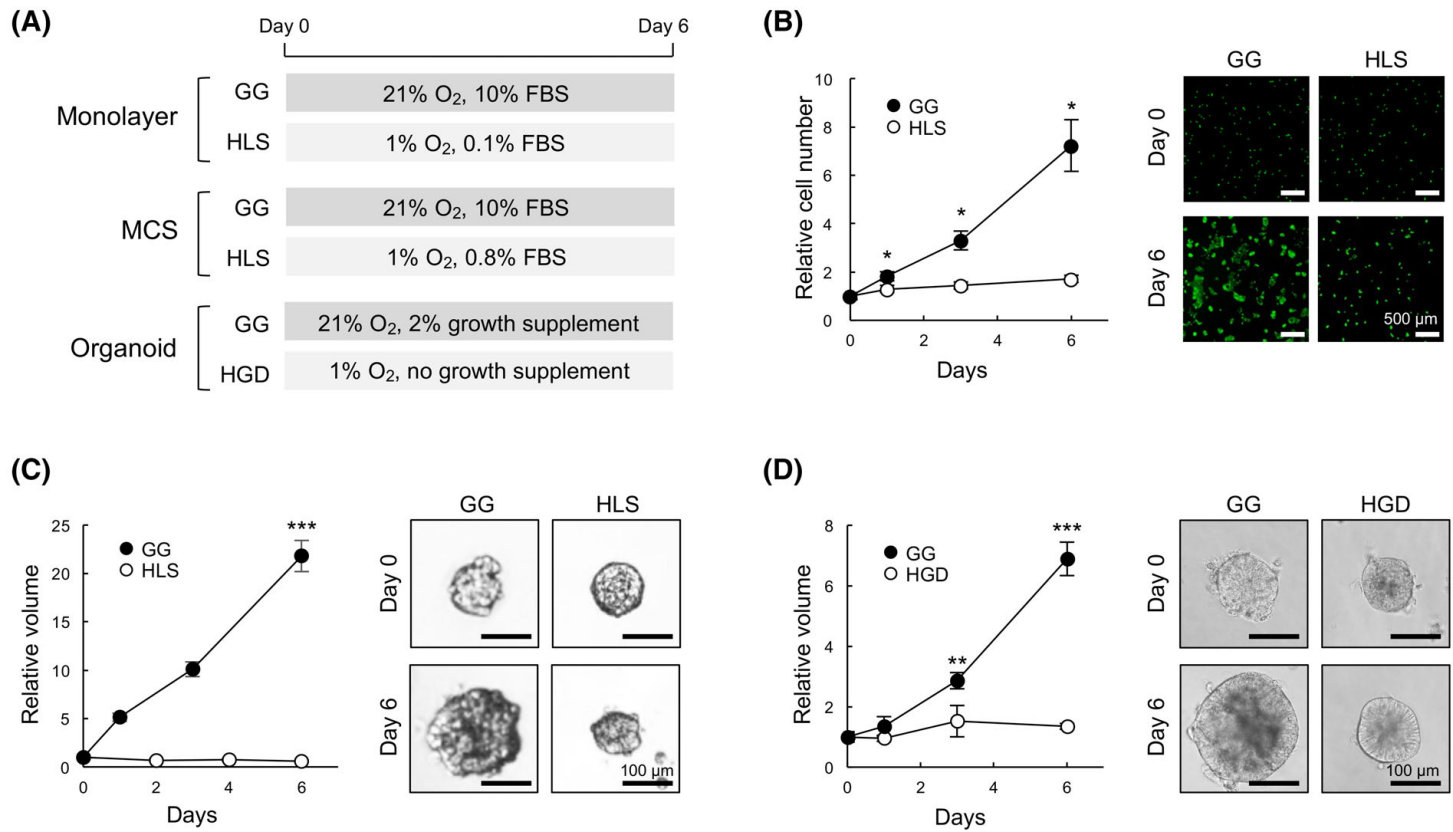
Preparation of quiescent cancer cell (QCC) models. (A) Schematic representation of the culture conditions for monolayer cells, multicellular spheroids (MCSs), and organoids. (B) Proliferation of H2228 monolayer cells cultured under general growth (GG) or hypoxic and low‐serum (HLS) conditions. Relative cell number (left) and representative fluorescence images (right) are shown. Living cells were labeled with CellTracker Green CMFDA. Scale bar, 500 μm, *n* = 3. (C) Growth of H2228 MCSs cultured under GG or HLS conditions. Relative spheroid volume (left) and representative bright‐field images (right) are shown. Scale bar, 100 μm, *n* = 30. (D) Growth of C45 organoids cultured under GG or hypoxic and growth supplement‐deprived (HGD) conditions. Relative spheroid volume (left) and representative bright‐field images (right) are shown. Scale bar, 100 μm, *n* = 4. Data are presented as means ± standard error of the mean (SEM). Statistical significance was determined using a two‐sided Student's *t*‐test. ***P < 0.001, **P < 0.01, *P < 0.05 versus GG.

DQ (Fig. 2A) showed markedly greater cytotoxicity toward quiescent than proliferating cells, with EC_50_ values of 0.29 ± 0.060 μm versus 2.8 ± 1.1 μm for H2228 monolayers, 1.6 ± 0.75 μm versus 23 ± 5.3 μm for H2228 MCSs, and 31 ± 7.6 μm versus > 50 μm for C45 organoids (Fig. 2B). In contrast, DQ showed minimal cytotoxicity toward normal WI‐38 fibroblasts (Fig. 2C). A derivative lacking one quinaldinium ring (DQ‐D, Fig. 2D) showed markedly reduced potency (Fig. 2E), suggesting that both rings are important for full activity.

**Fig. 2:**
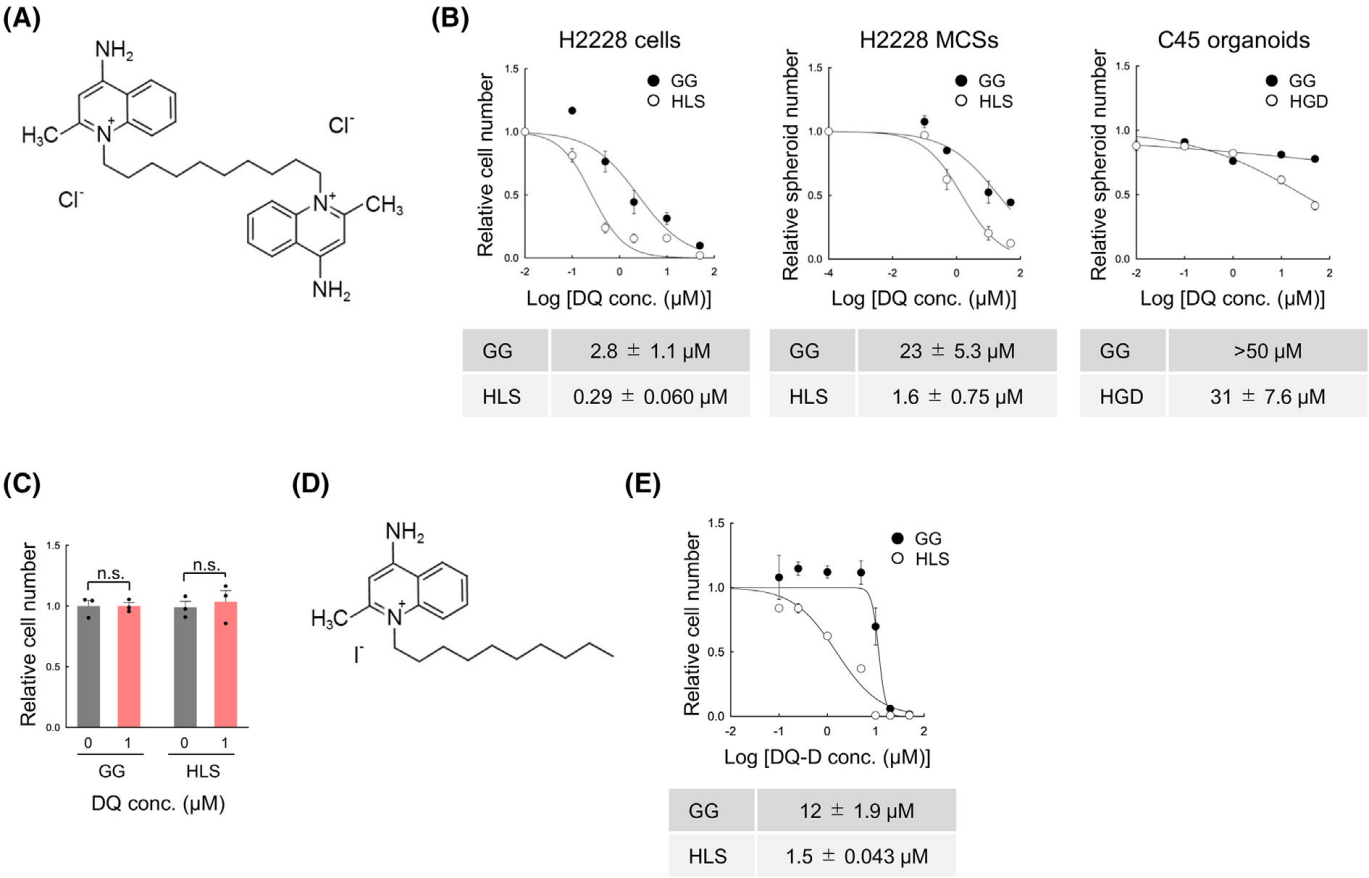
Dequalinium chloride (DQ) preferentially eliminates quiescent cancer cells (QCCs). (A) Chemical structure of DQ. (B) Cytotoxicity of DQ toward H2228 monolayer cells, H2228 multicellular spheroids (MCSs), and C45 organoids. H2228 cells cultured under general growth (GG) or hypoxic and low‐serum (HLS) conditions, H2228 MCSs cultured under GG or HLS conditions, and C45 organoids cultured under GG or hypoxic and growth supplement‐deprived (HGD) conditions were treated with serial concentrations of DQ. Relative cell number or relative spheroid number (top) and EC_50_ values calculated from the fitted curves (bottom) are shown. *n* = 5. (C) Cytotoxicity of DQ toward WI‐38 cells cultured under GG or HLS conditions. Relative cell number is shown. *n* = 3. n.s., not significant versus untreated cells. (D) Chemical structure of DQ derivative (DQ‐D). (E) Cytotoxicity of DQ‐D toward H2228 monolayer cells cultured under GG or HLS conditions. Relative cell number (top) and EC_50_ values calculated from the fitted curves (bottom) are shown. *n* = 3. Data are presented as means ± standard error of the mean (SEM). Statistical significance was determined using a two‐sided Student's *t*‐test.

### QCC‐selective killing by DQ correlates with ROS elevation

To investigate the basis of DQ selectivity, we first examined its effects on mitochondrial function. DQ reduced mitochondrial membrane potential and intracellular ATP levels to a similar extent in both quiescent and proliferating H2228 cells, suggesting that ATP depletion does not account for QCC selectivity (Fig. 3A,B).

**Fig. 3:**
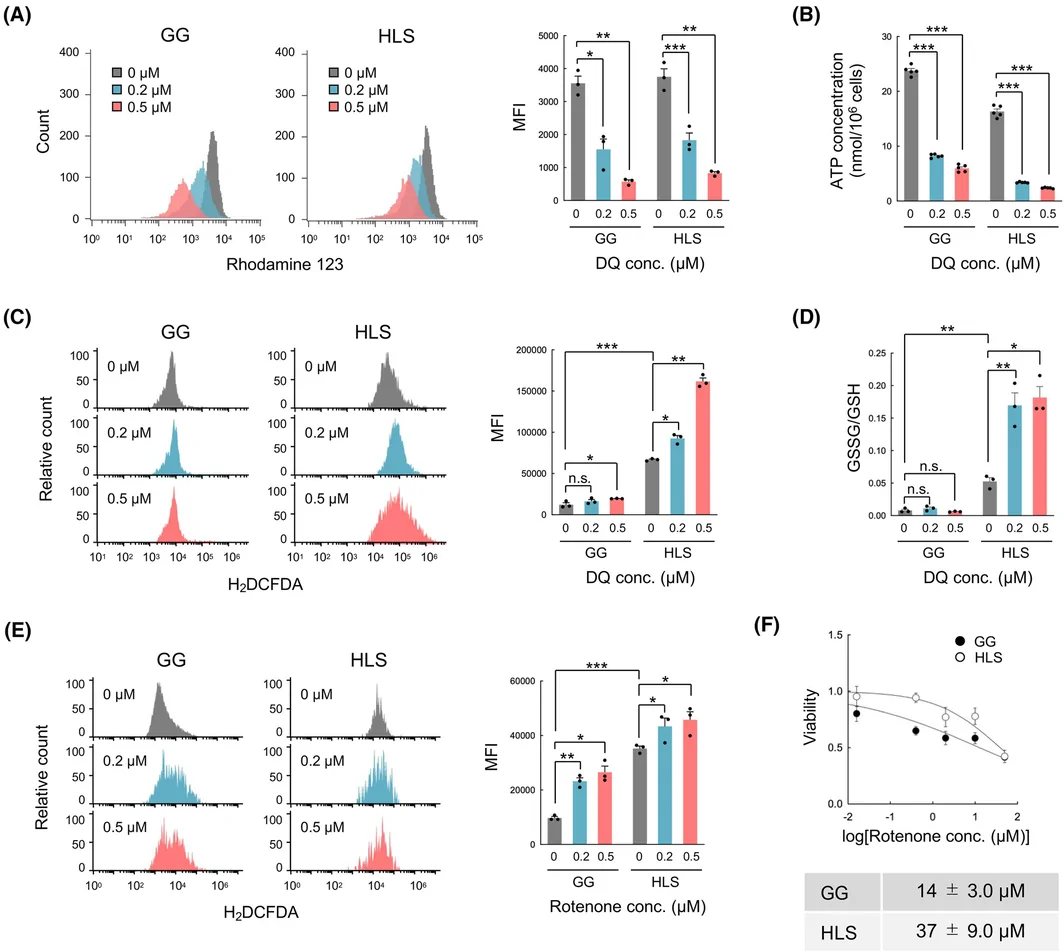
Quiescent cancer cell (QCC)‐selective cytotoxicity of dequalinium chloride (DQ) correlates with ROS elevation. (A) Mitochondrial membrane potential of DQ‐treated H2228 cells cultured under general growth (GG) or hypoxic and low‐serum (HLS) conditions. Cells stained with rhodamine 123 were analyzed by flow cytometry (FCM). Representative histograms (left) and mean fluorescence intensity (MFI) values (right) are shown. *n* = 3. (B) Intracellular ATP levels of DQ‐treated H2228 cells cultured under GG or HLS conditions. *n* = 3. (C) Intracellular ROS levels in DQ‐treated H2228 cells cultured under GG or HLS conditions. Cells stained with H_2_DCFDA were analyzed by FCM. Representative histograms (left) and MFI values (right) are shown. *n* = 3. (D) Redox status of DQ‐treated H2228 cells cultured under GG or HLS conditions. The ratio of oxidized glutathione (GSSG) to reduced glutathione (GSH) is shown. *n* = 3. (E) Intracellular ROS levels in rotenone‐treated H2228 cells cultured under GG or HLS conditions. Cells stained with H_2_DCFDA were analyzed by FCM. Representative histograms (left) and MFI values (right) are shown. *n* = 3. (F) Cytotoxicity of rotenone toward H2228 cells cultured under GG or HLS conditions. Relative cell number (top) and EC_50_ values calculated from the fitted curves (bottom) are shown. *n* = 3. Data are presented as means ± standard error of the mean (SEM). Statistical significance was determined using a two‐sided Student's *t*‐test. ***P < 0.001, **P < 0.01, *P < 0.05; n.s., not significant versus untreated cells.

We next assessed intracellular ROS levels and redox status. Even in the absence of treatment, quiescent H2228 cells exhibited higher basal ROS levels and a higher GSSG/GSH ratio than proliferating cells, consistent with intrinsic oxidative stress (Fig. 3C,D). Upon DQ treatment, ROS levels and GSSG/GSH ratios increased markedly in quiescent cells but only minimally in proliferating cells, indicating that QCCs carry a high intrinsic oxidative burden and are vulnerable to additional ROS stress.

To test whether the extent of ROS elevation determines cytotoxicity, we compared DQ with the mitochondrial complex I inhibitor rotenone. Rotenone caused a marked increase in intracellular ROS in proliferating H2228 cells but only a limited increase in quiescent cells (Fig. 3E), with correspondingly greater cytotoxicity toward proliferating cells (Fig. 3F). Thus, the quiescent cell selectivity of DQ is attributable to its unique ability to preferentially elevate ROS in quiescent cells.

### DQ induces ferroptosis in quiescent cells

Because elevated ROS can trigger multiple forms of cell death, we examined the pathways underlying DQ‐induced cytotoxicity. Flow cytometry showed that DQ primarily induced apoptotic cell death in proliferating cells, whereas quiescent cells underwent predominantly non‐apoptotic cell death (Fig. 4A). Although DQ did not activate caspase‐3/7 (Fig. 4B), it induced EndoG nuclear translocation (Fig. 4C), indicating EndoG‐mediated caspase‐independent apoptosis in proliferating cells.

**Fig. 4:**
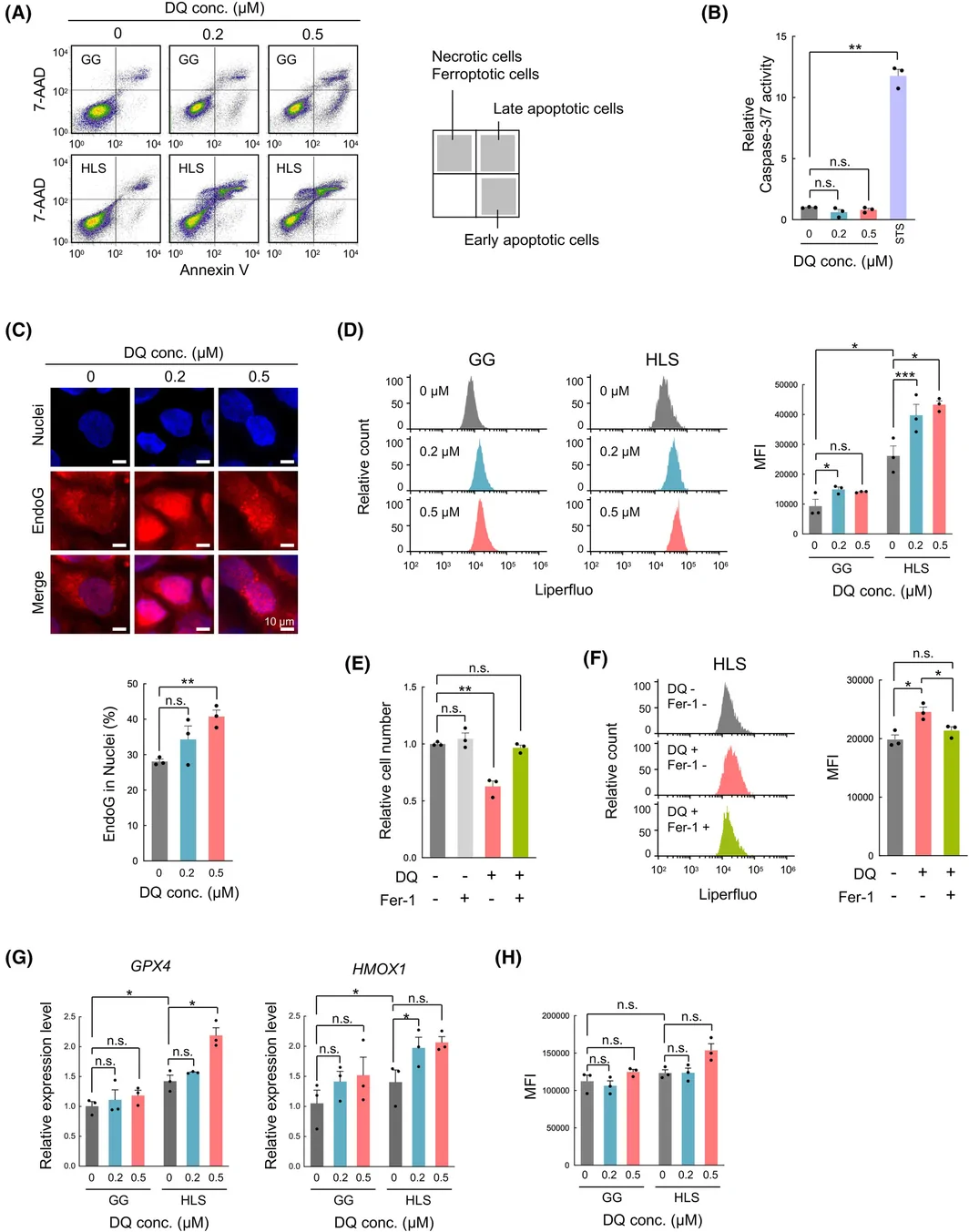
Dequalinium chloride (DQ) induces ferroptosis in quiescent cancer cells (QCCs). (A) Cell death analysis of DQ‐treated H2228 cells cultured under general growth (GG) or hypoxic and low‐serum (HLS) conditions. Cells stained with Annexin V and 7‐AAD were analyzed by flow cytometry (FCM). Representative plots are shown. (B) Caspase‐3/7 activity in DQ‐treated H2228 cells cultured under GG conditions. Relative activity is shown. STS, staurosporine. *n* = 3. (C) EndoG localization in DQ‐treated H2228 cells cultured under GG conditions. Representative fluorescence images (top) and the percentage of nuclear EndoG signal (bottom) are shown. EndoG, red; nuclei, blue. Scale bar, 10 μm. At least 20 cells per image were analyzed. *n* = 3. (D) Lipid peroxidation in DQ‐treated H2228 cells cultured under GG or HLS conditions. Cells stained with Liperfluo were analyzed by FCM. Representative histograms (left) and mean fluorescence intensity (MFI) values (right) are shown. *n* = 3. (E) Cytotoxicity of DQ in H2228 cells cultured under HLS conditions in the presence or absence of ferrostatin‐1 (Fer‐1). Relative cell number is shown. *n* = 3. (F) Lipid peroxidation in H2228 cells cultured under HLS conditions and treated with DQ and Fer‐1. Cells stained with Liperfluo were analyzed by FCM. Representative histograms (left) and MFI values (right) are shown. *n* = 3. (G) Relative mRNA levels of *GPX4* (left) and *HMOX1* (right) in H2228 cells cultured under GG or HLS conditions and treated with 0, 0.2, or 0.5 μm DQ for 3 days. Expression was normalized to *B2M*. B2M expression was stable across the tested conditions and was used as the internal control. *n* = 3. (H) Intracellular labile Fe^2+^ levels in H2228 cells cultured under GG or HLS conditions and treated with 0, 0.2, or 0.5 μm DQ for 3 days. Cells stained with FerroOrange were analyzed by FCM. MFI values are shown. *n* = 3. Data are presented as means ± standard error of the mean (SEM). Statistical significance was determined using a two‐sided Student's *t*‐test. ***P < 0.001, **P < 0.01, *P < 0.05; n.s., not significant versus untreated cells.

In quiescent cells, DQ markedly increased lipid peroxidation (Fig. 4D), a hallmark of ferroptosis, together with cell death (Fig. 4E). Co‐treatment with ferrostatin‐1, a lipid peroxidation inhibitor, completely blocked both DQ‐induced lipid peroxide accumulation and cell death (Fig. 4E,F), indicating that DQ induces ferroptosis selectively in quiescent cells.

To further characterize the redox response accompanying ferroptosis, we measured GPX4 and HMOX1 expression. Basal *GPX4* and *HMOX1* mRNA levels were higher under HLS than GG conditions and increased further after DQ treatment under HLS conditions (Fig. 4G), suggesting an enhanced transcriptional response to oxidative stress. Because ferroptosis is iron dependent and HO‐1 can influence iron availability, we next measured the intracellular labile Fe^2+^ pool. However, no significant differences were observed between GG and HLS conditions or following DQ treatment (Fig. 4H), indicating that the ferroptotic phenotype occurred without a detectable increase in the intracellular labile Fe^2+^ pool.

Together, these findings indicate that DQ eliminates quiescent H2228 cells, which intrinsically maintain high ROS levels and oxidative stress, by further increasing ROS and lipid peroxidation, thereby triggering ferroptotic cell death. In contrast, proliferating H2228 cells were less sensitive to DQ and underwent limited EndoG‐mediated apoptosis.

## Discussion

Our findings identify a metabolic and redox vulnerability in QCCs and demonstrate that DQ exploits this vulnerability to selectively induce ferroptosis in these cells. Hypoxic and nutrient‐limited conditions place QCCs at persistently elevated basal ROS levels, close to the threshold for oxidative collapse. Mitochondria‐targeted DQ further increases ROS levels beyond this threshold, triggering lipid peroxidation and ferroptosis in quiescent but not proliferating cells. These findings support redox destabilization as a potential strategy for targeting QCCs.

Prior studies have described DQ‐induced apoptosis or necrosis in various cancer cell types, but its ability to induce ferroptosis has not been evaluated. DQ has been reported to induce apoptosis through inhibition of XIAP in HeLa cervical cancer cells [31] and through ROS‐mediated caspase activation in PC‐3 prostate cancer cells [32], whereas in leukemia cell lines such as NB4 and K562 it induces necrosis through ATP depletion and excessive ROS generation [28]. These differences may reflect variations in mitochondrial stress and redox state across cell types. In proliferating H2228 cells, DQ elicited ROS levels sufficient to bypass caspase activation and promote EndoG‐mediated apoptosis, whereas in quiescent H2228 cells, which already exhibit elevated basal ROS and limited redox‐buffering capacity, DQ further increased ROS beyond the tolerance threshold, leading to lipid peroxidation and ferroptosis. In addition, many earlier studies predated the recognition of ferroptosis in 2012 and did not evaluate lipid peroxidation‐dependent cell death pathways [35]. Thus, our findings extend the current understanding of DQ cytotoxicity by showing that cellular metabolic state influences the mode of cell death.

As quiescent H2228 cells undergo ferroptosis as a result of increased oxidative stress, we compared DQ with other agents reported to target metabolically inactive cancer cells. For example, VLX600 has been shown to eliminate QCCs by impairing mitochondrial function [36]. However, VLX600 primarily acts as an iron chelator and does not induce substantial ROS production; instead, it promotes autophagy‐related cell death rather than ferroptosis [36, 37]. Thus, although both DQ and VLX600 disrupt mitochondrial metabolism, their modes of action differ. DQ amplifies mitochondrial ROS to exploit redox vulnerability, whereas VLX600 acts via ROS‐independent pathways. These differences indicate that mitochondrial‐targeted strategies can lead to divergent cell death outcomes depending on whether they induce oxidative stress.

Another important observation is the contrasting specificities of rotenone and DQ. While rotenone preferentially increased ROS levels in proliferating H2228 cells, DQ induced lethal ROS accumulation predominantly in quiescent H2228 cells. We previously reported that OXPHOS activity is higher in proliferating H2228 cells [11], which likely explains why rotenone, a mitochondrial complex I inhibitor, generates stronger oxidative stress in these cells through electron transport [38]. In contrast, DQ accumulates in mitochondria and disrupts membrane potential [27, 28], inducing mitochondrial permeability transition and triggering a ROS burst via ROS‐induced ROS release [39, 40]. This mechanism is less dependent on OXPHOS flux and instead exploits the limited redox‐buffering capacity of quiescent cells, leading to lipid peroxidation and ferroptosis. Thus, rotenone preferentially targets metabolically active proliferating cells, whereas DQ selectively eliminates QCCs by overwhelming their already stressed redox state.

Identification of ferroptosis as the predominant death pathway in QCCs highlights a therapeutic vulnerability that may be exploitable. Notably, DQ‐induced ferroptosis was accompanied by increased *GPX4* and *HMOX1* mRNA expression, suggesting activation of a compensatory response to oxidative stress. Despite HMOX1 induction, however, intracellular labile Fe^2+^ did not increase detectably, indicating that a sustained increase in the labile Fe^2+^ pool may not be required for DQ‐induced ferroptosis. One potential strategy is to combine mitochondrial perturbagens such as DQ with agents that sensitize cells to ferroptosis. SLC7A11‐mediated cystine uptake and glutathione peroxidase 4 (GPX4) activity are essential for maintaining glutathione levels and detoxifying lipid peroxides, and inhibition of either pathway promotes lipid peroxide accumulation and ferroptotic cell death [35, 41]. QCCs and drug‐resistant cancer cells have been reported to be highly sensitive to GPX4 inhibition [42, 43, 44], supporting the rationale for such combination approaches. In addition, the reversible H2228 QCC model used here may provide a useful platform for evaluating compounds that target residual cancer cells capable of recurrence. However, these conclusions are based on *in vitro* models, and *in vivo* studies will be required to establish the translational relevance of ferroptosis induction in QCCs.

In summary, we identified a redox‐based vulnerability in QCCs and showed that DQ selectively eliminates these cells by inducing ferroptosis, whereas proliferating cancer cells undergo caspase‐independent apoptosis. These findings establish the metabolic state as a determinant of death pathway selection and support redox‐targeted strategies for eliminating QCCs and preventing recurrence.

## Conflicts of interest

The authors declare conflicts of interest.

## Author contributions

**Kotaro Miyamoto:** formal analysis (lead), funding acquisition (supporting), investigation (lead), writing – original draft (lead), writing – review and editing (lead). **Shiori Sakai:** investigation (supporting). **Minori Endo:** investigation (supporting). **Terigele:** investigation (supporting). **Mizuho Niibori:** investigation (supporting). **Taiki Morita:** resources (equal). **Yu Goto:** investigation (supporting). **Arisa Yoshida:** investigation (supporting). **Tomohiro Tanaka**: investigation (supporting). Ning Lin: investigation (supporting). **Takahiro Kuchimaru:** writing – review and editing (supporting). **Hiroyuki Nakamura:** resources (equal), writing – review and editing (supporting). **Masahiro Inoue:** resources (equal), writing – review and editing (supporting). **Shinae Kizaka‐Kondoh:** conceptualization (equal), funding acquisition (supporting), supervision (equal), writing – review and editing (supporting). **Tetsuya Kadonosono:** conceptualization (equal), funding acquisition (lead), supervision (equal), writing – original draft (supporting), writing – review and editing (supporting).

## Data Availability

All data that support the findings of this study are available upon request from the corresponding author.
